# SimMS: a GPU-accelerated cosine similarity implementation for tandem mass spectrometry

**DOI:** 10.1093/bioinformatics/btaf081

**Published:** 2025-02-20

**Authors:** Tornike Onoprishvili, Jui-Hung Yuan, Kamen Petrov, Vijay Ingalalli, Lila Khederlarian, Niklas Leuchtenmuller, Sona Chandra, Aurelien Duarte, Andreas Bender, Yoann Gloaguen

**Affiliations:** Independent Consultant; Pangea Botanica Germany GmbH, Berlin 10623, Germany; Pangea Botanica Germany GmbH, Berlin 10623, Germany; Pangea Botanica Germany GmbH, Berlin 10623, Germany; Pangea Botanica Ltd, London WC1A 2AJ, United Kingdom; Wilde Ventures GmbH, Düsseldorf 40629, Germany; Pangea Botanica Germany GmbH, Berlin 10623, Germany; Independent Consultant; Pangea Botanica Germany GmbH, Berlin 10623, Germany; Pangea Botanica Germany GmbH, Berlin 10623, Germany

## Abstract

**Motivation:**

Untargeted metabolomics involves a large-scale comparison of the fragmentation pattern of a mass spectrum against a database containing known spectra. Given the number of comparisons involved, this step can be time-consuming.

**Results:**

In this work, we present a GPU-accelerated cosine similarity implementation for Tandem Mass Spectrometry (MS), with an approximately 1000-fold speedup compared to the MatchMS reference implementation, without any loss of accuracy. This improvement enables repository-scale spectral library matching for compound identification without the need for large compute clusters. This impact extends to any spectral comparison-based methods such as molecular networking approaches and analogue search.

**Availability and implementation:**

All code, results, and notebooks supporting are freely available under the MIT license at https://github.com/pangeAI/simms/.

## 1 Introduction

In the field of untargeted metabolomics, Tandem Mass Spectrometry (MS/MS) (Dettmer *et al.* 2007) is a well-established technique for identifying compounds within complex biological samples. A common workflow consists of comparing an unknown compound’s mass spectrum fragmentation pattern (“query”) against a database of known spectra (“reference”) in an effort to identify the unknown compound’s chemical composition (Bittremieux *et al.* 2022b).

Cosine similarity and its variants are popular methods facilitating MS/MS spectra comparison (Watrous *et al.* 2012, Schollée *et al.* 2017). Cosine similarity works by calculating a cosine of the angle between vectors of fragmentation intensities (“peaks”). Since an exact match between two fragmentation spectra is not practically possible due to measurement errors, finding the cosine score involves solving an *assignment problem* between the two sets of spectral peaks, with the goal of finding a valid matching of peaks (within *m/z* tolerance) that maximize the value of the cosine. Calculating the exact cosine similarity [Hungarian cosine (Munkres 1957)] is usually impractical for even moderate numbers of spectra. “Greedy” cosine similarity is an efficient approximation of the cosine similarity, which solves the assignment problem using a greedy heuristic (Huber *et al.* 2020). “Modified” cosine similarity is an extension of greedy cosine that uses the precursor mass as an additional input and has been shown to outperform greedy cosine in some cases (Watrous *et al.* 2012). Specifically, modified cosine has been shown to outperform a simple cosine similarity for the identification of non-identical but structurally related molecules (Bittremieux *et al.* 2022a), by applying a mass-shift to the MS2 spectra corresponding to the *m/z* value of their respective precursor ions. This approach is commonly used to match the spectra of analogue molecules.

Huber *et al.* (2020) also introduced MatchMS, an open-source python library for processing MS/MS. This library allows implementing easy-to-reproduce workflows to process raw mass spectral data into more useful forms (i.e. molecular networks). MatchMS conveniently implements all three types of cosine similarities. Unfortunately, while the MatchMS implementation is convenient, it is too slow for processing spectra on the scale of 10^11^ pairwise comparisons or more. Heavy workloads at such scale arise when processing results coming from a large study with public reference databases, and wider repository scale searches can simply not be achieved without heavy filtering and clean-up of the MS2 spectra to reduce the complexity of the task (Bittremieux *et al.* 2023). At such scales, MatchMS requires tens of CPU-days, necessitating a search for more efficient and robust approaches (Horai *et al.* 2010, Wang *et al.* 2016, Guijas *et al.* 2018).

To address this issue, Harwood *et al.* (2023) introduced BLINK, which approximates the greedy cosine by first blurring the query and the reference spectral peaks, transforming them into sparse matrices, and then calculating a sparse outer product between the two, to directly compute the score matrix. This effectively side-steps solving the peak assignment problem, allowing BLINK significant speed improvements over MatchMS. Unfortunately, with tolerance values larger than 0.01 BLINK rapidly loses accuracy (Harwood *et al.* 2023). Other attempts to accelerate spectra matching have been made, however they rely on reformulating the scoring methods. These methods are orthogonal to our approach (Li and Fiehn 2023, Mongia *et al.* 2024).

In the current report, we speed up Cosine calculation from an engineering point of view. We leverage the fact that cosine calculation is readily parallelizable and rewrite both greedy and modified cosine algorithms using CUDA (Vingelmann and Fitzek 2020) into a custom, highly optimized GPU kernel that, depending on underlying GPU hardware, can process spectra up to 1000 times faster than MatchMS. We show that the approach exactly replicates MatchMS results and supports a much wider tolerance range without compromising accuracy. Furthermore, we distribute the kernel into a user-friendly Python package that can act as a drop-in replacement for respective MatchMS cosine similarity classes. All code, results, and notebooks supporting this report are freely available under the MIT license at https://github.com/pangeAI/simms/.

## 2 Results

**Figure 1.**
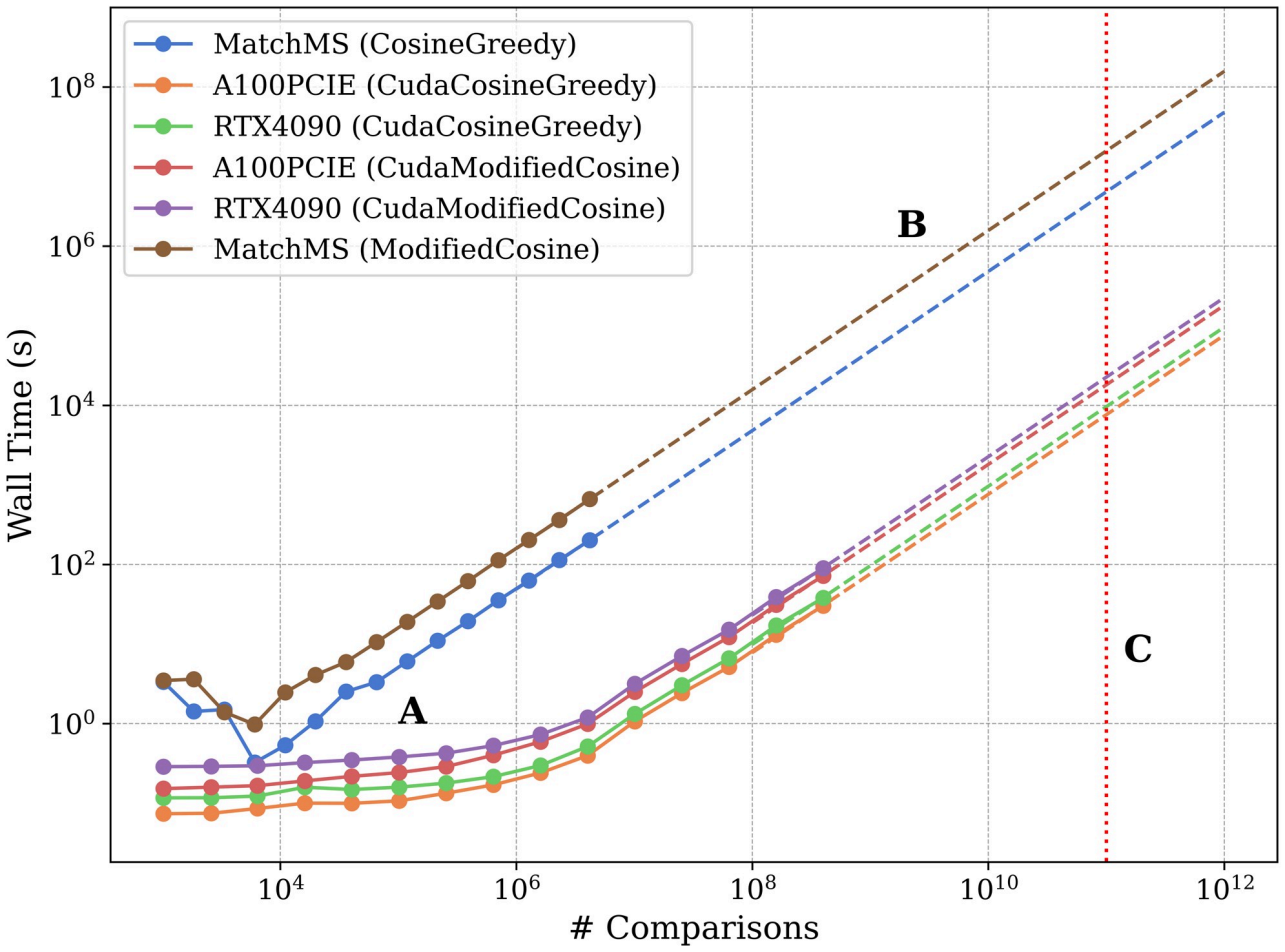
Cosine scoring runtimes for GNPS subsets with default parameters, with different GPUs and methods. Marker dots (A) represent real measurements, and dashed lines (B) are their linear extrapolations. (C) is a marker of 10^11^ comparisons, and represents our target processing goal as defined above. Notably, all approaches seem to have a similar computational time complexity.

We found that using the Cosine kernel implementation presented in this work with a single NVIDIA A100 GPU for calculations (Choquette *et al.* 2021) is ∼1000× faster than using MatchMS for both greedy and modified cosine similarities, as shown in Fig. 1. Calculating greedy cosine similarity at the scale of 100 000 queries paired with 1 million reference spectra takes an estimated 9 weeks with MatchMS, in contrast to only 2 h using our kernel.

Modified cosine similarity is slower than greedy cosine (also shown in Fig. 1), independent of hardware used, since the steps required to calculate the modified cosine score includes all steps required for calculating the greedy cosine score (Watrous *et al.* 2012).

**Figure 2.**
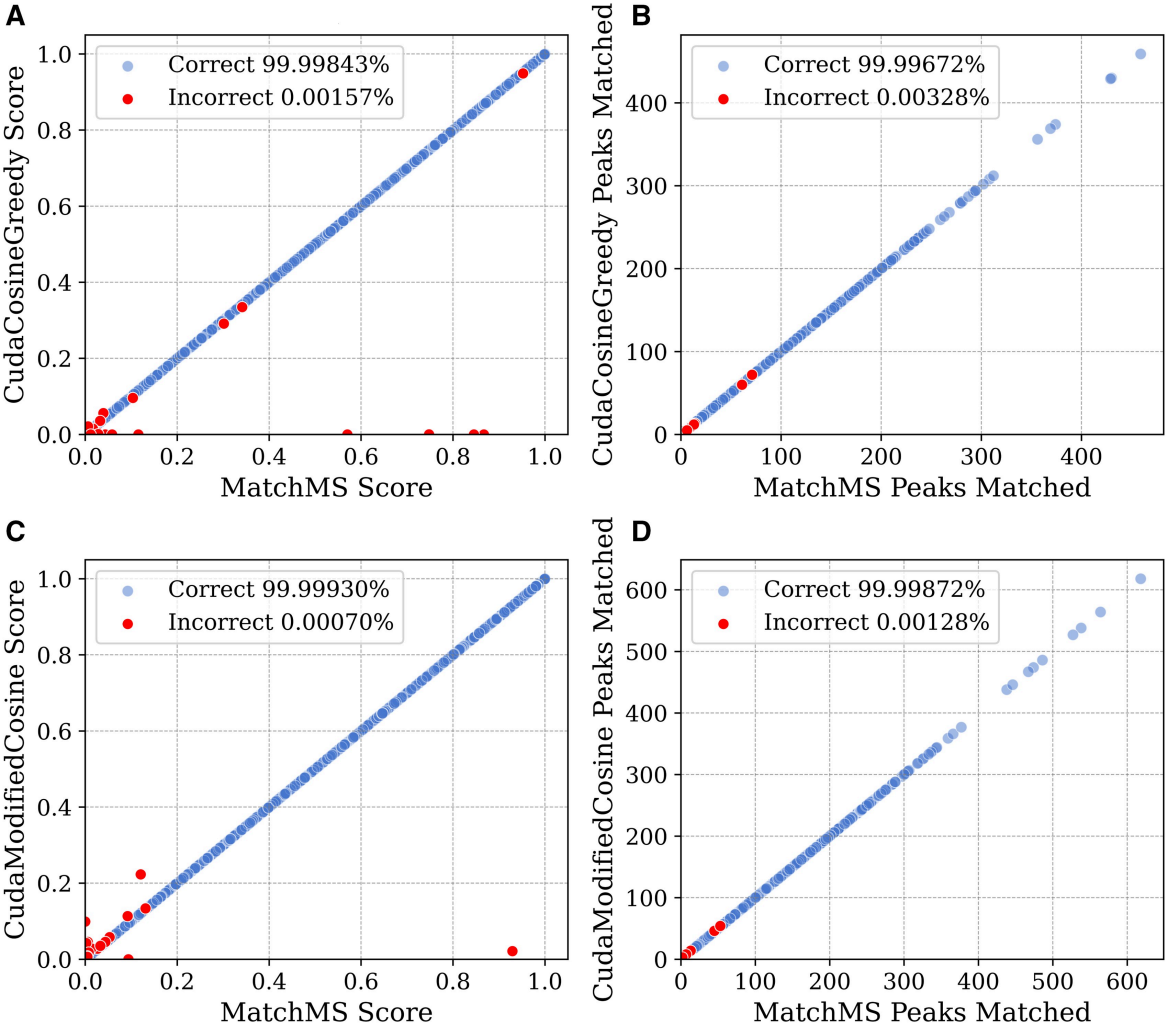
Direct comparison of scores of 4.1M random GNPS spectra pairs, illustrating SimMS error patterns. Red dots denote incorrect (absolute error > 0.001) SimMS outputs. (A) and (B) show greedy cosine results between SimMS and MatchMS, performed at float32 precision. In (C) and (D), we compare modified cosine results at float64 precision. Occasional large errors in (A) and (C) are caused by spectra with very few (one or two) large peaks.

We performed a direct comparison of results for predicted scores and the number of matches, as shown in Fig. 2. We found that for default parameters, the kernel and MatchMS results are within ±0.001 of each other 99.99% of the time. Since the kernel is algorithmically equivalent to its MatchMS counterpart, the few errors that do occur stem from peaks that are almost exactly tolerance distance apart. In other words, when paired with MatchMS, these peaks appear to be within the tolerance distance, but processing them with a GPU changes their binary floating-point representation, which is enough to make them appear outside the tolerance distance. When a spectrum has a single very intense peak, such binary representation changes can result in large score errors. The score comparison plots in Fig. 2A and C panels show these kinds of errors in the bottom right.

We find that the rate of incorrect scores is lower than the rate of incorrect match counts. The reason is that an off-by-one match count does not always result in a significantly different score. An extra or missing small peak can contribute <0.001 to the score, while changing the match count by 1.

**Figure 3.**
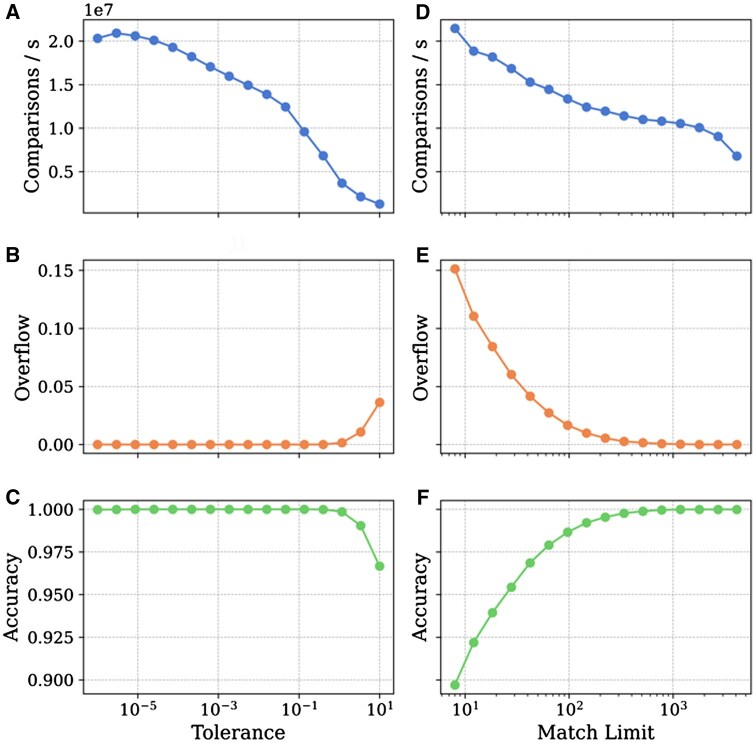
Key metrics as a function of changing tolerance (A)–(C) and match limit (D)–(F). Plots A and D show the average number of comparisons per second. “Overflow” is the proportion of scores that are returned with the overflow flag. Accuracy is the proportion of scores that are within ±0.001 of MatchMS.

In Fig. 3, we can see how changing the tolerance and match limit influences SimMS performance. Reducing tolerance or match limit by two orders of magnitude approximately doubles the processing speed, while overflow rate is inversely proportional to the match limit. The accuracy rate in Fig 3C and F is approximately equal to the 1 − overflow rate.

**Figure 4.**
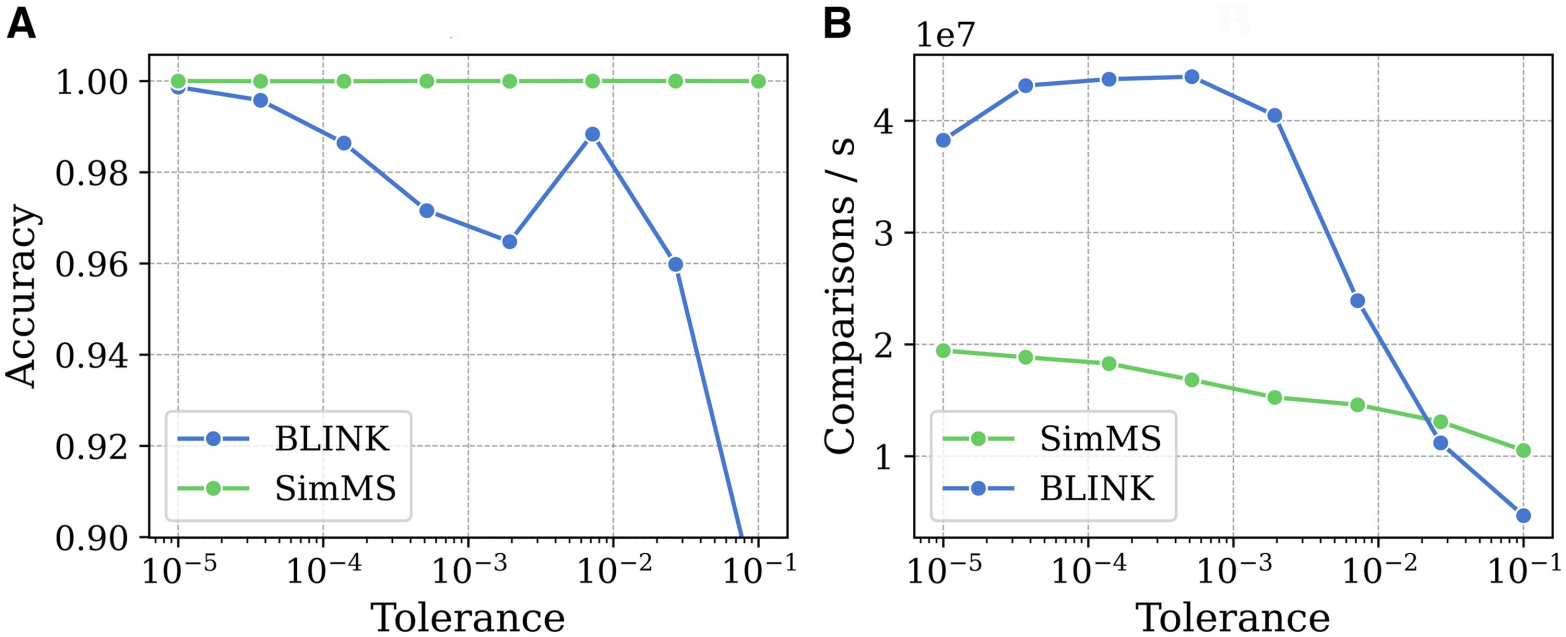
Accuracy and speed comparison of SimMS and BLINK. Accuracy is the proportion of scores that are within ±0.001 of MatchMS reference, and the speed is the number of comparisons per second.

We compared our approach with BLINK across a range of tolerance values, as shown in Fig. 4. Within the investigated tolerance window of 10^−5^ to 10^−1^, SimMS shows perfect accuracy with the MatchMS reference. BLINK has 99.88% accuracy when tolerance is 10^−5^, and 89.88% when tolerance is 10^−1^. We find that BLINK is twice as fast as SimMS when tolerance is lower than 10^−3^. As tolerance increases, the performance of BLINK quickly degrades. BLINK and SimMS have approximately the same speed around 0.03 tolerance. At 0.1 tolerance, SimMS is nearly 2.5× times faster than BLINK. Overall, our approach offers a stable and more accurate solution than BLINK over a wide range of tolerance values.

## 3 Methods

### 3.1 Implementation

We used as input a list of references and queries, denoting their respective lengths as **R** and **Q**. For both lists, consecutive spectra are grouped into batches of size **B**, the last batch contains leftover spectra and padding, as needed.

**Figure 5.**
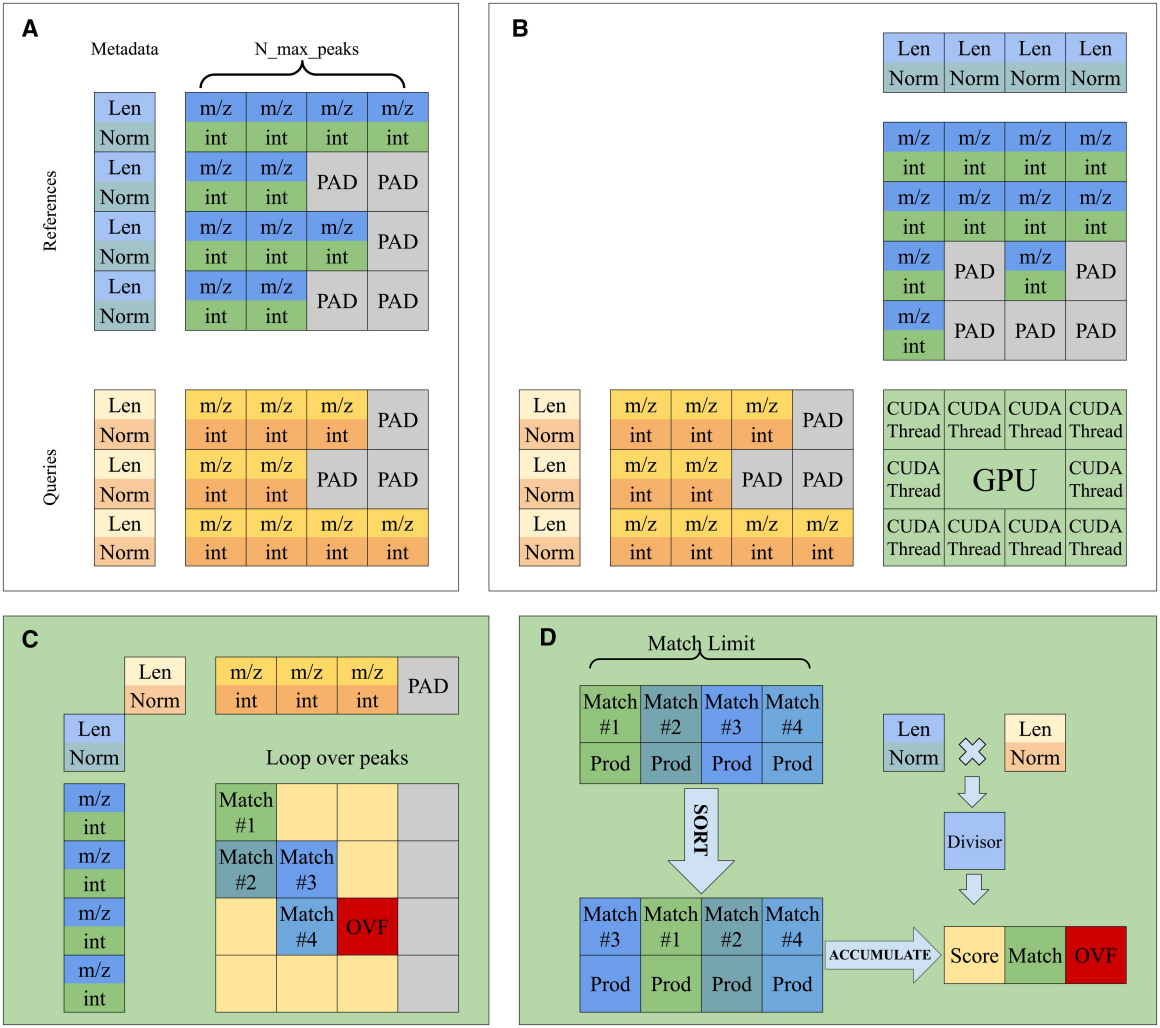
Overview of processing on GPU. In a first step (A), we pack all spectra and metadata in 3D and 2D tensors, respectively. In a second step (B), we align the spectra with the GPU grid. Steps (C) and (D) happen within a single GPU thread. In (C), we accumulate potential matching peaks up to the given match limit. In the last step (D), we sort, deduplicate and reduce matched peaks, returning three values.

Inside each batch, all spectra are concatenated into a single $R^{2,B,M}$ tensor, where **M** is the number of peaks in the longest spectrum in that batch, **B** is the batch size (Fig. 5A). A batch contains stacked peak m/z and intensity values in the first dimension. Spectra that are shorter than **M** are padded with zeros. Spectra that are larger than **M** are truncated with an argument $N_{\max\;\mathrm{peaks}}$.

The “metadata” tensor is created alongside the spectra batch. The metadata tensor contains the length, cosine norm, and, in case of modified cosine, the precursor m/z values. Metadata tensor is a $R^{K,B}$ tensor, where **K** is either 4 or 6, and **B** is the batch size. For greedy cosine, dimension **K** is 4 and consists of lengths of reference and query spectra and norms of reference and query spectra. In case of modified cosine, the metadata additionally contains precursor m/z values for reference and query spectra. For batches corresponding to leftover spectra, we pad the resulting empty space with zeros.

The full similarity matrix of size R×Q is infeasible to store in GPU memory. Therefore, processing is done block-by-block. The full R×Q grid is split into B×B sized, non-overlapping blocks. In total, this results in $\mathrm{ceil}\left(\frac{R}{B}\right)\times \mathrm{ceil}\left(\frac{Q}{B}\right)$ number of blocks. Since we have three return values for each comparison: the score, the number of matches, and the overflow flag, an output $R^{3,B,B}$ tensor is allocated on the GPU per block. A pair of batches of spectra, and their respective metadata tensors are transferred to the GPU. The tolerance, *m/z* power, intensity power, $N_{\mathrm{match}\;\mathrm{limit}}$ and $N_{\max\;\mathrm{peaks}}$ are supplied to the kernel as compilation-time constants. At this point the kernel is launched.

The kernel is written using Numba (Lam *et al.* 2015). Inside the kernel, a single CUDA thread is assigned to calculate the cosine score between one reference spectrum and one query spectrum from the supplied batches. The computing power of SimMS stems from the fact that modern GPUs can process tens of thousands of threads simultaneously, allowing us to process each block in a fraction of a second.

The kernel itself consists of three main stages (Fig. 5C and D). First, pairs of peaks within tolerance are collected. A maximum of $N_{\mathrm{match}\;\mathrm{limit}}$ pairs are collected. If the number of pairs exceeds this limit, an overflow flag is raised and the collection stops early. Next, the pairs are sorted by the value of the product of the intensities of paired peaks. Finally, the ordered pairs are deduplicated and accumulated into an unnormalized cosine score. An auxiliary boolean array is used to mark each accumulated peak, to avoid duplicate contributions to the score. As a final step, the two norms from metadata are multiplied to obtain the normalizing constant, which divides the unnormalized score to produce the final cosine score (Fig. 5D). In the case of the modified cosine score calculation, the initial stage accumulates ${2N}_{\mathrm{match}\;\mathrm{limit}}$ pairs instead of $N_{\mathrm{match}\;\mathrm{limit}}$. The first half of these pairs is collected exactly as described above. The second half, however, is collected only after applying a mass shift to the *m/z* values. Specifically, for each peak in both spectra, the precursor *m/z* value is subtracted from the fragment *m/z* value of that peak. The rest of the calculation proceeds as previously described.

After the execution, the block output tensor contains three results for every reference/query pair in the batch. These are: score, number of matches, and overflow (binary flag). Each block is then concatenated together in order to form the full R×Q similarity matrix. In case of processing a very large set of references and queries (larger than 100 000), the required memory to store the full similarity matrix as a dense array is impractically large. Additionally, we find that most of the scores are lower than 0.5. For such cases we use “sparse” implementation, where the similarity matrix is filtered to discard all results with score below a user-defined “sparse threshold” and then store the remaining entries as a sparse matrix in DOK format.

Finally, we concatenate all the results and return them, either as a dense array or as a sparse DOK matrix.

### 3.2 Hardware

All of our kernel and MatchMS experiments were performed on rented Vastai (Vast.ai) instances. Preferably, we rented instances with at least 16GB RAM, eight or more CPUs, and at least a single RTX4090 GPU. Performance of the original MatchMS algorithm is independent from GPU, while our kernel performance heavily depends on it—NVIDIA A100 GPU usually outperforms RTX4090, which in turn outperforms older GPUs.

### 3.3 Interoperability

We have taken care to make the SimMS package fully compatible with MatchMS. During the implementation of the kernel, we also discovered that Cosine Greedy scores from MatchMS were semi-randomly fluctuating when run on different CPU hardware, causing the exact MatchMS results to be unrepeatable. We later patched this bug, and from version 0.24.0 onwards, the patch has been merged into the core MatchMS package. Correction details are available at https://github.com/matchms/matchms/pull/596.

The full code is available on GitHub at https://github.com/PangeAI/simms under an MIT license. An archive of the code is available on Zenodo at https://doi.org/10.5281/zenodo.14678214.

## 4 Discussion and future work

We presented a new python package that uses GPU parallelism to accelerate mass spectra cosine similarity calculation. GPU-accelerated cosine similarity algorithms enable fast searching in large repositories of MS data (e.g. MassIVE GNPS) without sacrificing the accuracy of the original algorithms. Further accelerating the processing via multi-GPU processing is left as a topic of future research.

## Supplementary Material

btaf081_Supplementary_Data
