# Supplementary material for: SimMS: a GPU-accelerated cosine similarity implementation for tandem mass spectrometry
Source: Bioinformatics. 2025 Feb 20;41(3):btaf081. doi: 10.1093/bioinformatics/btaf081 (PMC11886821; doi:10.1093/bioinformatics/btaf081)
Supplement: btaf081_Supplementary_Data [file btaf081_supplementary_data.docx]

**SimMS: A GPU-Accelerated Cosine Similarity implementation for Tandem Mass Spectrometry**

Tornike Onoprishvili^1^, Jui-Hung Yuan^2^, Kamen Petrov^2^, Vijay Ingalalli^2^, Lila Khederlarian^3^, Niklas Leuchtenmuller^4^, Sona Chandra^2^, Aurelien Duarte^1^, Andreas Bender^2^, Yoann Gloaguen^2,*^

^1^Independent consultant, ^2^Pangea Botanica Germany GmbH, Hardenbergstrasse 32, 10623 Berlin, Germany, ^3^Pangea Botanica Ltd, 15 Southampton Pl, London WC1A 2AJ, United Kingdom, ^4^Wilde Ventures GmbH, In der Rehwiese 3, 40629 Düsseldorf, Germany

*To whom correspondence should be addressed.

# Supplementary information

## The kernel algorithm

def kernel(reference, query, info) -> float, int, bool:

matches = array(max_size=match_limit)

score_norm = get_rnorm(info) * get_qnorm(info)

# 1. Collect peaks

for r, q in cartesian(reference, query):

if matches.has_space():

if abs(r - q) < tol:

matches.append((r, q))

else:

overflow = 1; break

# 2. Sort peaks

matches = sorted(matches)

# 3. Filter

visited = array(max_size=n_max_peaks, fill=False)

score, num_matches = 0, 0

for r, q in matches:

if not (visited(r) or visited(q)):

score += get_peak_product(r, q)

num_matches += 1

visited[r], visited[q] = True, True

score = score/score_norm

return score, num_matches, overflow

**Algorithm S1. Kernel pseudo-code.**
